# Efficacy of eravacycline in difficult-to-treat (DTR) *Acinetobacter baumannii* bacteraemia in ICU: a case report

**DOI:** 10.1093/jac/dkaf347

**Published:** 2025-09-23

**Authors:** Monica Melchio, Federica Portunato, Antonio Vena, Elisa Porcile, Monica Centanaro, Matteo Bassetti

**Affiliations:** Division of Infectious Diseases, Department of Health Sciences, University of Genova, Genoa, Italy; Division of Infectious Diseases, IRCCS Ospedale Policlinico San Martino, L.go R. Benzi, 10, Genoa 16132, Italy; Division of Infectious Diseases, IRCCS Ospedale Policlinico San Martino, L.go R. Benzi, 10, Genoa 16132, Italy; Division of Infectious Diseases, Department of Health Sciences, University of Genova, Genoa, Italy; Division of Infectious Diseases, IRCCS Ospedale Policlinico San Martino, L.go R. Benzi, 10, Genoa 16132, Italy; Anesthesiology and Intensive Care Unit, IRCCS Ospedale Policlinico San Martino, Genoa, Italy; Anesthesiology and Intensive Care Unit, IRCCS Ospedale Policlinico San Martino, Genoa, Italy; Division of Infectious Diseases, Department of Health Sciences, University of Genova, Genoa, Italy; Division of Infectious Diseases, IRCCS Ospedale Policlinico San Martino, L.go R. Benzi, 10, Genoa 16132, Italy

*Acinetobacter baumannii* is a Gram-negative pathogen responsible for nosocomial infections associated with high mortality rates, primarily due to the development of multiple resistance mechanisms against most antibiotics. In ICUs, the overall mortality rate can reach up to 54% in ventilator-associated pneumonia (VAP), bacteraemia and septic shock.^1^

Traditionally, treatment options for DTR *A. baumannii* infections have been suboptimal, often involving combination regimens that include high doses of ampicillin–sulbactam, carbapenems, colistin or tigecycline. However, these regimens have been associated with high rates of renal and hepatic toxicity as well as subtherapeutic concentrations at the infection site.^2^

In recent years, new antimicrobial agents with improved tolerability with activity against *A. baumannii* have been approved, including cefiderocol, eravacycline and durlobactam/sulbactam.^3^ However, the latter is not yet available in Europe.

Here, we report a case of DTR *A. baumannii* bacteraemia successfully managed with eravacycline monotherapy.

At the end of 2024, a male patient in his forties was transferred from a secondary hospital to the ICU of our university hospital in Northern Italy due to haemorrhagic shock and respiratory failure, requiring endotracheal intubation. His past medical history included obesity (BMI 34), diabetes and recent hospitalization for haematemesis secondary to oesophageal varices, which led to the diagnosis of metabolic dysfunction-associated steatohepatitis cirrhosis.

On admission, rectal swab screening revealed colonization by carbapenem-resistant *A. baumannii* (OXA-23 producer).

His respiratory status rapidly worsened, and chest CT demonstrated bilateral consolidative pneumonia. Bronchoalveolar lavage (BAL) was performed, and rapid molecular test identified the presence of *A. baumannii*, confirmed by conventional culture. Susceptibility results are shown in Table 1.

**Table 1.** Microbiological results obtained by molecular techniques and conventional culture methods

	First episode		Second episode	
Molecular diagnostic^a^	BAL fluid: *Acinetobacter calcoaceticus-baumannii complex*		Blood culture: *Acinetobacter calcoaceticus-baumannii complex*	

	Concentration: >10E7 cp/mL			
	No resistance mechanism detected^b^		No resistance mechanism detected^b^	
Conventional culture	BAL fluid culture: *Acinetobacter baumannii* (10E5 UFC)		Blood culture: *Acinetobacter baumannii*	

	Antibiogram		Antibiogram	
Antibiotic	Interpretation	MIC	Interpretation	MIC
Amikacin	R	>32	R	>32
Ciprofloxacin	R	>2	R	>2
Gentamicin	R	>8	R	>8
Colistin	S	1	R	4
Imipenem/relebactam	R	>8	R	>8
Meropenem	R	>8	R	>8
Meropenem/vaborbactam	—	>32	—	>32
Piperacillin/tazobactam	—	>64	—	>64
Tigecycline	—	4	—	4
Cefiderocol	—	23 mm	—	18 mm
Eravacycline				0.25

^a^Molecular diagnostics were performed using the FilmArray Pneumonia + Panel (BioFire, bioMérieux^®^) on the BAL fluid sample and the FilmArray Blood Culture Identification Panel (BioFire, bioMérieux^®^) on the positive blood culture.

^b^The molecular panels are able to detect the following carbapenemase genes: IMP, KPC, NDM, OXA-48 like and VIM.

In accordance with current IDSA guidelines, VAP treatment was initiated with ampicillin–sulbactam (27 g/24 h) and cefiderocol (2 g every 6 h—extended infusion), dosed for his glomerular filtration rate (GFR) of 123 mL/min.^3^ After 6 days with limited improvement, colistin was added (loading dose 9 million IU, then 4.5 million IU every 12 h), leading to gradual clinical recovery.

Following 17 days of therapy, antibiotics were discontinued. With optimized haemodynamic status through fluid balance reduction, the patient remained stable for nearly 2 weeks.

He then developed new-onset fever and haemodynamic deterioration. Blood cultures were performed, and one of four blood culture bottles turned positive within 2 h. Rapid molecular testing identified *A. baumannii*, prompting re-initiation of ampicillin–sulbactam (27 g/24 h) and cefiderocol (2 g every 8 h; GFR 65 mL/min). Conventional cultures confirmed the isolate in blood, and the same pathogen was also recovered from broncho-aspirate (Table 1).

Given the recent antibiotic exposure and updated susceptibility profile—showing a reduced inhibition zone diameter for cefiderocol—eravacycline susceptibility testing was performed, yielding a MIC of 0.25 mg/L. After 48 h of combination therapy with ampicillin–sulbactam and cefiderocol, haemodynamic stability was achieved, and treatment was switched to eravacycline monotherapy (1 mg/kg every 12 h). No dose adjustment was made, as he had moderate hepatic impairment (Child–Pugh Class B), and current literature does not consistently recommend dose reduction in this setting.^4^ A blood culture obtained 24 h after the initiation of eravacycline was negative, and clearance of bacteraemia was confirmed by two additional blood culture sets performed at 48 and 72 h of therapy. Eravacycline was continued for a total of 7 days, leading to fever resolution and clinical improvement.

Over the following 3 weeks, his condition steadily improved, allowing transfer to a rehabilitation unit and, after a further month, the patient was discharged home. At 6-month follow-up, he remained in good health and continued regular outpatient monitoring for his liver disease.

Our case demonstrates successful clinical and microbiological cure of DTR *A. baumannii* bacteraemia with eravacycline monotherapy in a critically ill patient.

Eravacycline is a next-generation tetracycline characterized by a favourable pharmacokinetic and pharmacodynamic profile.^5^ Although *in vitro* studies suggest activity against *A. baumannii*,^6^ real-world data remain limited, and published results have been conflicting.^7–11^

A few retrospective non-controlled studies have reported encouraging outcomes, with clinical cure rates ranging from 70% to 95%, including cases involving *A. baumannii* infections.^7–9,11^ Nonetheless, the small sample sizes, limited susceptibility data and variability in the use of monotherapy versus combination regimens complicate the interpretation of these real-life experiences.

Worth mentioning is the cohort study by Scott *et al*.,^10^ which compared eravacycline (used as part of a combination regimen in 70% of cases) with the previously best available therapy for DTR *A. baumannii* pneumonia. In this study, the eravacycline group exhibited a higher 30-day mortality rate than controls (33% versus 15%, *P* = 0.048), with particularly poor outcomes observed in the four patients treated with eravacycline with *A. baumannii* bacteraemia.

In contrast to the findings of Scott *et al.*, our case shows that eravacycline monotherapy can achieve both clinical and microbiological cure in selected patients with DTR *A. baumannii* bacteraemia. While a single case cannot provide definitive evidence, it offers valuable real-world insight into a possible therapeutic option. Further well-designed studies with larger patient populations are needed to clearly define eravacycline’s role, particularly in bacteraemic presentations.

The patient was informed about the conduct and publication of this case study and provided written and verbal informed consent. The study was carried out in accordance with the Declaration of Helsinki.
